# Rapid Bioassay-Guided Isolation of Antibacterial Clerodane Type Diterpenoid from *Dodonaea viscosa* (L.) Jaeq.

**DOI:** 10.3390/ijms160920290

**Published:** 2015-08-27

**Authors:** Muhammad Khurram, Linda A. Lawton, Christine Edwards, Marcello Iriti, Abdul Hameed, Murad A. Khan, Farman A. Khan, Shafiq ur Rahman

**Affiliations:** 1 Department of Pharmacy, Shaheed Benazir Bhutto University, Sheringal, Dir Upper 18000, Pakistan; E-Mail: shafiq@sbbu.edu.pk; 2 School of Pharmacy and Life Sciences, the Robert Gordon University, Aberdeen AB25 1HG, UK; E-Mails: l.lawton@rgu.ac.uk (L.A.L.); c.edwards@rgu.ac.uk (C.E.); 3 Department of Microbiology, Quaid-i-Azam University, Islamabad 45320, Pakistan; E-Mail: ahameed@qau.edu.pk; 4 Department of Agricultural and Environmental Sciences, Milan State University, Milan 20133, Italy; 5 Centre for Interdisciplinary Research in Basic Sciences, International Islamic University, Islamabad 44000, Pakistan; E-Mail: abdul.hameed@iiu.edu.pk; 6 Department of Chemistry, Kohat University of Science & Technology, Kohat 26000, Pakistan; E-Mail: dr.murad@kust.edu.pk; 7 Department of Chemistry, Shaheed Benazir Bhutto University, Sheringal, Dir Upper 18000, Pakistan; E-Mail: farmanali@sbbu.edu.pk

**Keywords:** *Dodonaea viscosa*, preparative HPLC, XTT assay, clerodane diterpenoid

## Abstract

Plant extracts are complex matrices and, although crude extracts are widely in use, purified compounds are pivotal in drug discovery. This study describes the application of automated preparative-HPLC combined with a rapid off-line bacterial bioassay, using reduction of a tetrazolium salt as an indicator of bacterial metabolism. This approach enabled the identification of fractions from *Dodonaea viscosa* that were active against *Staphylococcus aureus* and *Escherichia coli*, which, ultimately, resulted in the identification of a clerodane type diterpenoid, 6β-hydroxy-15,16-epoxy-5β, 8β, 9β, 10α-cleroda-3, 13(16), 14-trien-18-oic acid, showing bacteriostatic activity (minimum inhibitory concentration (MIC) = 64–128 µg/mL) against test bacteria. To the best of our knowledge, this is the first report on antibacterial activity of this metabolite from *D. viscosa.*

## 1. Introduction

The global presence and rise of antibiotic resistant pathogens and decline in antibiotic drug discovery programs by pharmaceutical companies are prompting the scientific community to search for new and also re-examine old sources of bioactive chemicals in order to identify potential drugs [1]. Medicinal plants are an area under focus since their secondary metabolites encompass a significant number of drugs used in current therapeutics and there is no doubt in their potential as the source of new medicines [2].

*Dodonaea viscosa* (L.) Jaeq. is an evergreen shrub in Pakistan and is locally known as Ghawraskay (Pushto). It is traditionally used to treat a wide range of medical conditions from colds to malaria [3,4], and crude extracts have been shown to be active against bacterial, viral, fungal and protozoal pathogens [5,6,7]. However, despite numerous phytochemical investigations, only a single compound, hautriwaic acid, with moderate antibacterial activity has been characterized [8].

Isolation and characterization of bioactive molecules from crude plant extracts or fractioned extracts are challenging tasks. This is mainly due to the presence of a large number of metabolites with close physicochemical properties with often complementing bioactivities. However, in order to standardize them, their separation is necessary. Chromatographic methods have revolutionized the separation of such complex mixtures and the combination of chromatography with spectroscopic and spectrophotometric methods such as mass spectroscopy, UV, NMR *etc.* has further eased the task. In this context, preparative HPLC has shown to be an essential tool for producing high quality natural products and if strict quality control is undertaken, the approach ensures reliable screening results [9,10].

Currently, cell viability assays make use of one of several tetrazolium salts in a microplate format. Metabolically active cells reduce the tetrazolium salts and result in coloured formazan products that can be simply read by a plate reader, thus offering rapid records on the metabolic status of the cell [11]. XTT (2,3-bis(2-methoxy-4-nitro-5-sulfophenyl)-2H-tetrazolium-5-carboxanilide) is a tetrazolium salt that does not require the additional solubilisation step as required in the 3-(4,5-dimethylthiazol-2-yl)-2,5-diphenyltetrazolium bromide (MTT) assay and yields a reduced formazan product that is water soluble [11]. The XTT assay is exploited in determination of antimicrobial activity of simple plant extracts [12,13]. This study describes the combination of automated preparative HPLC in combination with an XTT bioassay for rapid identification of antibacterial fractions and derived active compound from *D. viscosa*.

## 2. Results and Discussion

### 2.1. Fractionation of Plant Material

Liquid-liquid fractionation of the crude aqueous extract of *D. viscosa* resulted in 75 g of an *n*-hexane fraction (H), 70 g of a dichloromethane fraction (D), 92 g of an ethyl acetate fraction (E), 81 g of an *n*-butanol fraction (B) that spontaneously solidified, and 126 g of thick suspension of an aqueous fraction (A).

### 2.2. Preliminary Antibacterial Screening

Extracts of *D. viscosa* derived from hexane and ethyl acetate were active against *E. coli* (the National Collection of Industrial, Marine and Food Bacteria (NCIMB) 8797) and *S. aureus* (NCIMB 6571), whereas the butanol extract was only active against *S. aureus* (Table 1). Previous studies on *D. viscosa* fractions showed bacteriostatic activity against Gram positive bacteria, but not Gram negative such as *E. coli* [5,6]; our results indicated that the hexane and ethyl acetate fractions inhibited *E. coli*. These opposite findings may be due to differences in the plant material, extraction protocols and test strains used. As there is growing interest in the generation of libraries of simplified extracts prior to screening [9,10], our approach aimed to select those extracts which exhibited antibacterial activity and use preparative HPLC with the XTT assay for rapid screening of a large number of fractions.

**Table 1:** Antibacterial activity (average zones of inhibitions in mm ± SD) of crude solvent fractions determined by disk diffusion assay.

*D. viscosa*	Test Bacteria	
Fraction	*E. coli* (NCIMB 8797)	*S. aureus* (NCIMB 6571)
Hexane (H)	12.5 ± 0.5	11.4 ± 0.5
Dichloromethane (D)	0.0	0.0
Ethyl acetate (E)	9.7 ± 0.3	9.0 ± 0.2
Butanol (B)	0.0	10.0 ± 0.2
Aqueous (A)	0.0	0.0
Ciprofloxacin	31.0 ± 0.5	–
Clarithromycin	–	28.6 ± 0.5

Ciprofloxacin (10 µg/disk); Clarithromycin (15 µg/disk) (positive controls); Methanol (negative control).

### 2.3. Optimization of XTT (2,3-Bis(2-methoxy-4-nitro-5-sulfophenyl)-2H-tetrazolium-5-carboxanilide) Bioassay

Ciprofloxacin (50 µg/mL) and clarithromycin (25 µg/mL) inhibited the test strains of *E. coli* and *S. aureus*, respectively, after 24 h incubation in the XTT assay, as shown by the decreased absorbance. The effect of ciprofloxacin on *E. coli* was evident after 2 h incubation, with absorbance in the treated cells at 0.042 compared to the solvent control at 0.098. However, 24 h incubation was necessary to obtain an evident result on the effect of clarithromycin on *S. aureus*; therefore 24 h incubation was selected as incubation period.

### 2.4. Generic Preparative HPLC-XTT Bioassay

**Figure 1:**
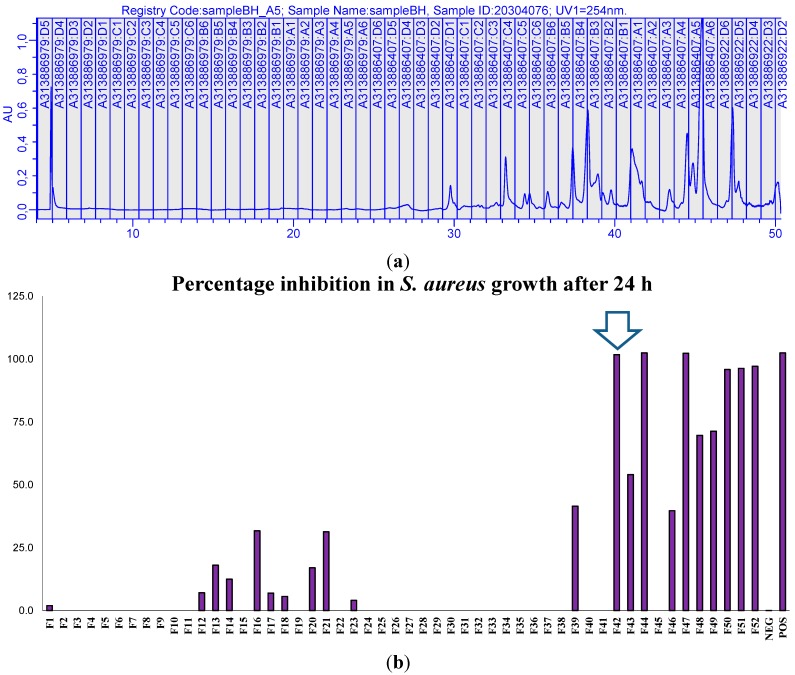

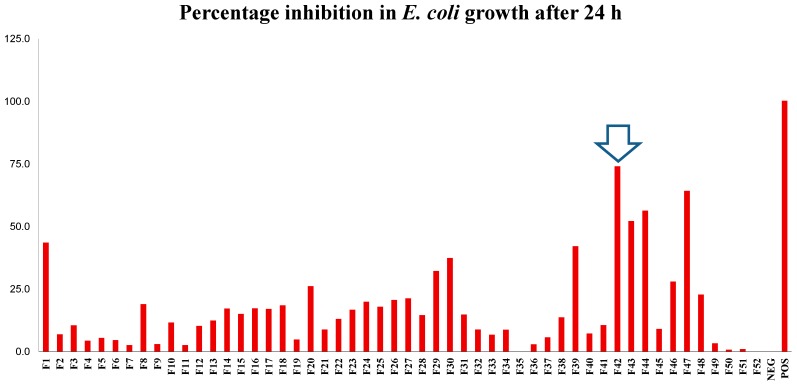
Preparative separation of *n*-hexane extract of *D. viscosa* using generic gradient and volume-based fractionation (**a**), percentage inhibition (**b**) of *S. aureus* (NCIMB 6571), percentage inhibition (**c**) of *E. coli* (NCIMB 8797), where NEG represented the negative control of 10% (*v*/*v*) dimethyl sulfoxide in phosphate buffer saline (DMSO/PBS) and the positive controls (POS) of clarithromycin (25 µg/mL) and ciprofloxacin (50 µg/mL), respectively (*n* = 2 ± SD). Fraction 42 (arrow) contained high purity, bioactive compound. XTT (2,3-bis(2-methoxy-4-nitro-5-sulfophenyl)-2H-tetrazolium-5-carboxanilide) Bioassay results are depicted in Figure S1.

The use of solvents such as methanol and water along with a volume-based fractionation strategy was necessary as the active component(s) may not contain a chromophore. For the extract, a total of 52 primary fractions were collected and each fraction was evaluated against the two test bacteria using the XTT assay. No bioactivity was detected in the fractions from the ethyl acetate extract of *D. viscosa*, despite the activity against both bacteria reported in disk diffusion assay (Table 1), and they were not investigated further. The butanol extract of *D. viscosa* yielded three fractions (18, 19 and 20) with moderate antibacterial activity, *i.e.*, 50%–75% reduction in bacterial growth. In contrast, six fractions from the hexane extract inhibited *S. aureus*, with this antibacterial activity resulting in higher than 75% reduction in bacterial activity (Figure 1).

**Figure 2:**
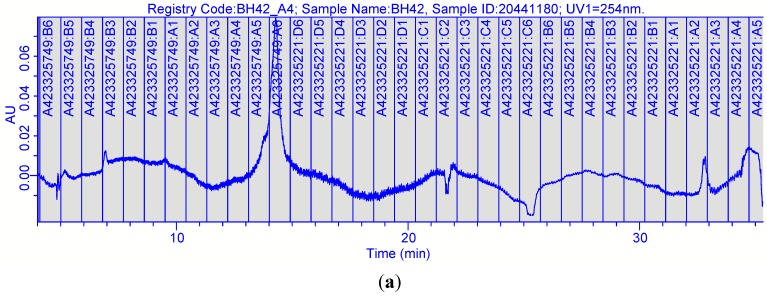

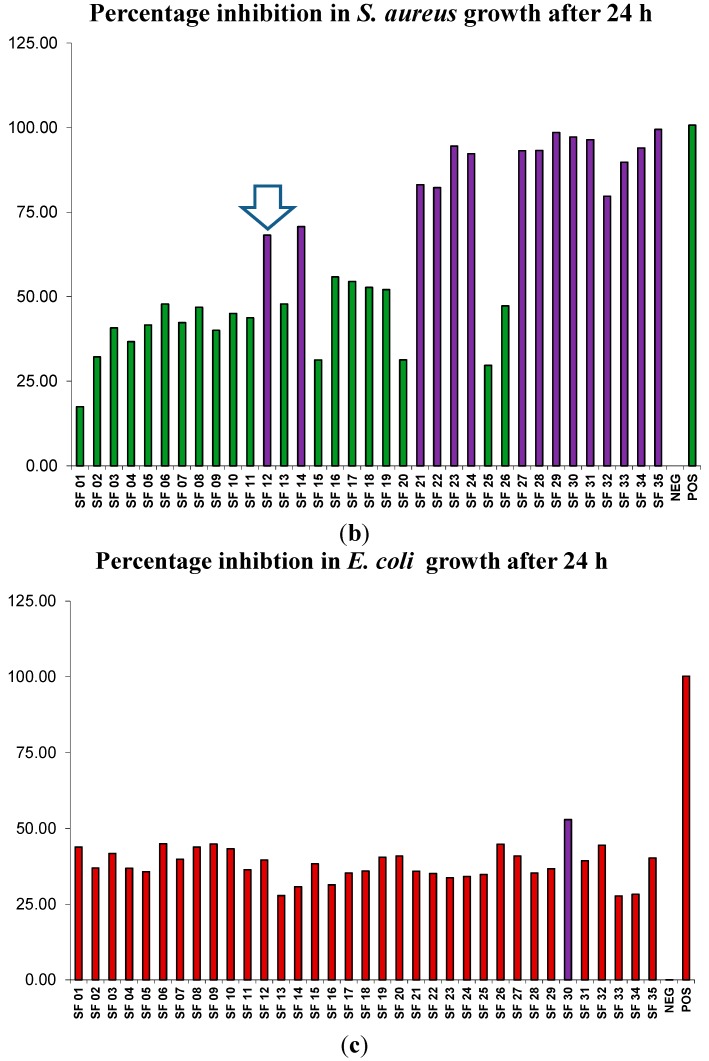
Separation and fractionation of bioactive fraction 42 from *n*-hexane extract of *D. viscosa* using polarity focused gradient (**a**) percentage inhibition (**b**) of *S. aureus* (NCIMB 6571), percentage inhibition (**c**) of *E. coli* (NCIMB 8797), where NEG represented the negative control of 10% (*v*/*v*) DMSO/PBS and POS the positive controls of clarithromycin (25 µg/mL) and ciprofloxacin (50 µg/mL) respectively, (*n* = 2 ± SD). Fraction 12 (arrow) contained high purity, bioactive compound. XTT Bioassay results are depicted in Figure S2.

Figure 2 shows the data from the preparative HPLC and the results of XTT assay, correlating with the active fractions to the corresponding peaks in the chromatogram and indicating the hydrophobic components of the fractions. Even if fractions with antibacterial activity were clearly evident, the majority of fractions enhanced bacterial viability (fractions with no inhibition), most likely due to additional carbon sources.

### 2.5. Focused Gradients-XTT Bioassay

Nine bioactive fractions from *D. viscosa* were further purified using focused gradients and each of the separated fractions were screened by the rapid XTT assay. The five primary fractions from the hexane extract from *D. viscosa* resulted in 22 fractions, which inhibited growth of *S. aureus*, and out of which 18 were as active as the positive control clarithromycin.

**Figure 3:**
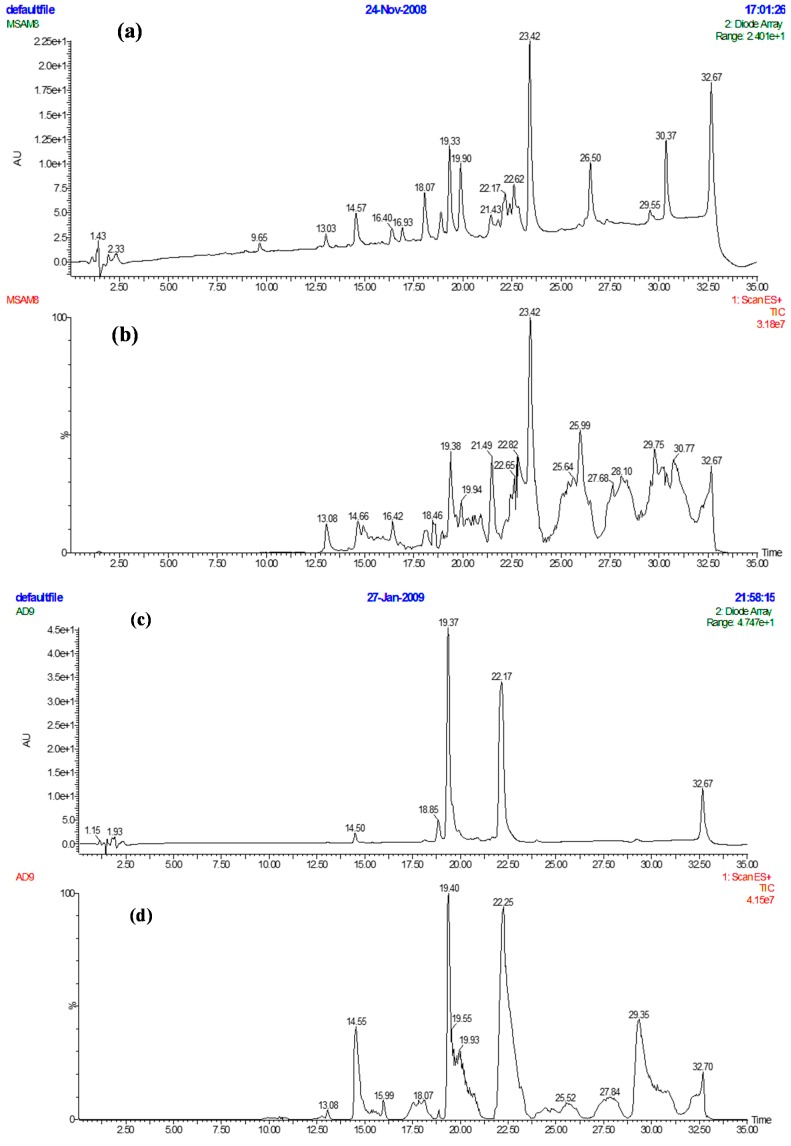

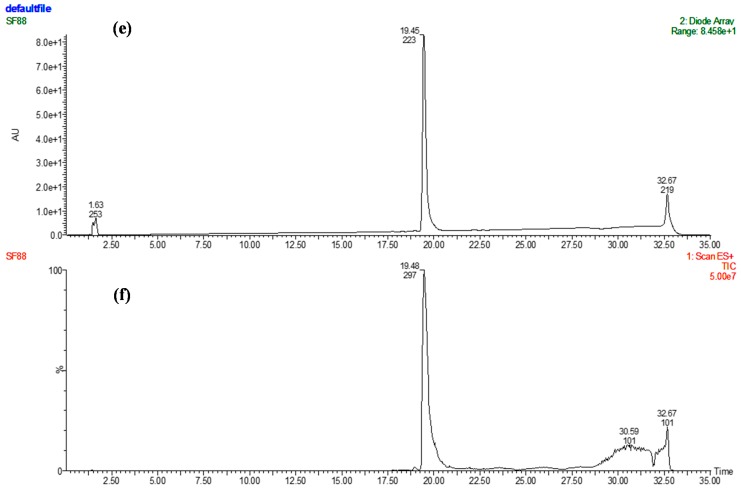
(**a**) Separation (photodiode array detector (PDA) chromatogram) of crude hexane *D. viscosa* extract by reversed phase HPLC; (**b**) Separation (MS chromatogram) of crude hexane *D. viscosa* extract by reversed phase HPLC; (**c**) bioactive fraction 42 (PDA chromatogram) from generic preparative separation; (**d**) bioactive fraction 42 (MS chromatogram) from generic preparative separation; (**e**) bioactive fraction 12 (PDA chromatogram) from polarity focused separation; (**f**) bioactive fraction 12 (MS chromatogram) from polarity focused separation.

The combination of preparative HPLC with the XTT assay enabled the rapid isolation of bioactive fractions from plant extracts. In addition, after purification, information on purity and physicochemical properties of bioactive fractions were obtained by LC/MS analysis. Data from the XTT assay (Figure 2) and LC/MS analysis (Figure 3) led to focusing on one compound from *D. viscosa*. Though this information alone was not sufficient for full characterization of the compound, it provided essential information for monitoring, purifications and obtaining higher amounts of compounds, thus avoiding unnecessary bioassays.

The prime objective of these combination bioassays was to identify fractions with antibacterial activity. At the preliminary stages (fractions and sub-fractions level) quantification was not considered necessary with the reason that synergism and/or potentiation may exist between the respective metabolites that exist in a particular fraction, therefore, during initial screening, fractions were analysed on a qualitative basis. Activity was quantified when a purified bioactive metabolite was obtained.

**Figure 4:**
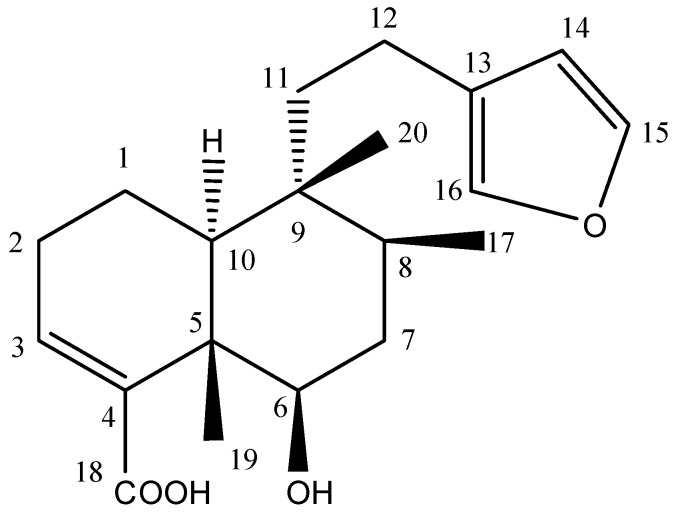
Chemical structure of compound **1** from *D. viscosa*.

The antibacterial compound **1** (Figure 4) from *D. viscosa* found in fractions H42 and sub-fraction 12 was purified and its structure was elucidated using the latest spectroscopic techniques including 1D and 2D NMR spectra (Table 2). Compound **1** was isolated as a white crystalline solid and showed single absorption in UV at 217.7 nm. Its molecular formula was established as C_20_H_28_O_4_ by its EI-MS molecular ion peak at *m*/*z* 332.1879 (calculated (calcd.) for C_20_H_28_O_4_, 332.2013). Other major peaks appeared at *m*/*z* 314.9 (loss of H_2_O) and at *m*/*z* 270.4 (loss of H_2_O and CO_2_). The ^1^H-NMR spectrum displayed signals assignable to an unsaturated decalin system as δ = 6.86 (1H, dd, *J* = 2.9, 4.6 Hz (H-3)), 1.22 (3H, s, H-17), 0.87 (3H, d, *J* = 6.8 Hz (H-18)) and 0.76 (3H, s, H-20). Furthermore, the signals of a furane moiety appeared at δ = 6.30 (1H, brs, H-14), 7.38 (1H, t, *J* = 0.8 Hz, H-16) and 7.27 (1H, brs, H-15) respectively. In addition, the ^1^H-NMR spectrum showed signals at δ = 3.64 (1H, dd, *J* = 10.8 Hz, 5.1 Hz (H-6)), which was assigned to Carbon 6 in the structure. ^1^H-^1^H COSY (Correlation Spectroscopy) experiments confirmed the C-6 proton by vicinal coupling cross peaks to both methylene protons of C-7. ^13^C-NMR spectrum revealed twenty carbon signals including six olefinic carbons at δ_C_ 143.2 (C-15), 111.1 (C-14), 141.9 (C-4), 140.6 (C-3), 139 (C-16) and 125.9 (C-13) while the acid carbonyl carbon (C-18) resonated downfield at δ_C_ 174.1. In the DEPT (Distortionless Enhancement by Polarization Transfer) experiment among 20 carbons, three methyl, five methylene, seven methine and five quaternary carbons were distinguished. Further correlations in structure were made through HSQC (Heteronuclear Single Quantum Coherence) and HMBC (Heteronuclear Multiple Bond Correlation) experiments (Table 2). The spectral data was in complete agreement with 6β-hydroxy-15,16-epoxy-5β, 8β, 9β, 10α-cleroda-3, 13(16), 14-trien-18-oic acid, the known compound in literature [14].

**Table 2:** ^13^C- and ^1^H**-**NMR spectral data (methanol-*d*_4_) and ^1^H/^13^C correlations of compound **1**.

C. No	^13^C-NMR (δ)	Multiplicity DEPT	^1^H-NMR (δ)	^1^H/^13^C (HMBC) Connectivity
1	17.5	CH_2_	α1.75 (m) β1.65 (m)	H-3 (6.86)
2	27.4	CH_2_	2.33 (m)	H-3 (6.86), H-10 (1.40)
3	140.6	CH	6.86 (dd, 2.9, 4.6)	H-2 (2.33), H_α_-1 (1.75), H_β_-1 (1.65)
4	141.9	C	–	H-3 (6.86), H-6 (3.64), H-17 (1.22)
5	45.1	C	–	H-3 (6.86), H-17 (1.22)
6	75.2	CH	3.64 (dd, 5.1, 10.8)	H-17 (1.22)
7	36.4	CH_2_	1.60 (m)	–
8	34.4	CH	1.76 (m)	H-18 (0.87), H-19 (0.76)
9	39.3	C	–	H-18 (0.87)
10	46.2	CH	1.40 (bd, 12.0)	H-19 (0.76)
11	39.0	CH_2_	1.61 (m)	H-10 (1.40), H-20 (0.76)
12	18.0	CH_2_	2.30 (m)	H-19 (0.76)
13	125.9	C	–	H-12 (2.30), H-14 (6.30), H-15 (7.38), H-16 (7.27)
14	111.1	CH	6.30 (bs)	H-15 (7.38), H-16 (7.27)
15	143.2	CH	7.38 (t, 1.8)	H-14 (6.30), H-16 (7.27)
16	139.0	CH	7.27 (bs)	H-12 (2.30), H-14 (6.30), H-15 (7.38)
17	15.2	CH_3_	0.87 (d, 6.8)	–
18	174.1	C	–	–
19	16.1	CH_3_	1.22 (s)	H-6 (3.64)
20	17.4	CH_3_	0.76 (s)	–

### 2.6. MIC (Minimum Inhibitory Concentration) and MBC (Minimum Bactericidal Concentration) of Compound **1**

**Figure 5:**
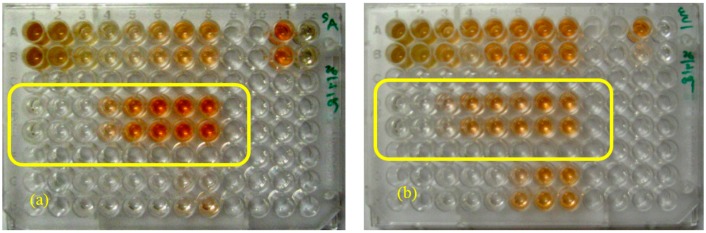
MICs of compound **1** (in yellow rectangle) isolated from *D. viscosa* against *S. aureus* (**a**) and *E. coli* (**b**).

The antibacterial activity of the compound **1** was quantified by evaluating the MIC (minimum inhibitory concentration) and MBC (minimum bactericidal concentration) against *S. aureus* (NCIMB 6571) and *E. coli* (NCIMB 8797). The results indicated a higher activity against *S. aureus* compared to *E. coli*, thereby suggesting it to be more effective against Gram-positive bacteria (Table 3 and Figure 5). The compound was crystalline, white coloured and soluble in 10% (*v*/*v*) DMSO in PBS solution and, hence, no color interference was reported with the XTT assay.

**Table 3:** MIC (minimum inhibitory concentration) and MBC (minimum bactericidal concentration) of compound **1** isolated from *D. viscosa* against *S. aureus* and *E. coli*.

Bacterial Susceptibility Assays	Compound 1		Clarithromycin		Ciprofloxacin	
	*S. aureus*	*E. coli*	*S. aureus*	*E. coli*	*S. aureus*	*E. coli*
MIC (μg/mL)	64	128	25	–	–	25
MBC (μg/mL)	128	256	50	–	–	50

10% (*v*/*v*) DMSO in PBS served as negative control.

A few Clerodane furanolactones and diterpenoid derivatives were previously identified in the liverwort *Scapania nemorae* [15], *Solidago* spp. [16] and in *Aristeguetia* spp. [17]. However, to the best of our knowledge, this is the first report on its antibacterial activity. Previously, five clerodane diterpenoids isolated from *Pulicaria wightiana* showed antibacterial activity against *Bacillus subtilis*, *B. sphaericus*, *S. aureus*, *Klebsiella aerogenes* and *Chromobacterium violaceum* [18]. The results obtained from the bio-guided isolation of furanyl clerodanoic acid in *D. viscosa*, as well as the antimicrobial activity of hautriwaic acid, a *D. viscosa* component, reported to be active against *B. subtilis*, *S. aureus* and *E. coli* [8], support the possible use of this plant for the treatment of infectious diseases in the traditional systems of medicines. The application of the XTT bioassay in identification of bioactive metabolite and quantification of its antibacterial activity using standard test bacteria provides an approach that may be utilized in similar kinds of experiments. Since, in case of plant extracts, the commonly utilized assays (agar well diffusion and disk diffusion assays) that only highlight the antimicrobial activity of extracts or their fractions do not demonstrate the actual potential of that particular plant, more detailed standardized assays are essential. Moreover, the potential of isolated compounds can be easily quantified through MIC assays using the XTT bioassay-based approach.

The isolated compound belongs to isoprenoids that are known to possess antimicrobial activity. In general, Gram-positive bacteria are more susceptible to these compounds, as confirmed by our results. Putative mechanisms of action may include cell membrane disruption, alteration in the cell membrane fluidity and permeability, increased susceptibility to antibiotics, and disturbances in the respiration chain due to modification of membrane-bound protein localization [19]. Moreover, isoprenoids may also be considered for adjuvant therapy with conventional antimicrobials, in order to reduce, delay or impair antibiotic resistance. Studies on antibiotic activity of clerodane type diterpenoid on other microorganisms are still in progress, and further investigations on its possible mechanism(s) of action will be planned and designed.

## 3. Experimental Section

### 3.1. Chemicals

Dichloromethane, *n*-hexane, ethyl acetate and *n*-butanol were obtained from Merck (Darmstadt, Germany). Acetonitrile and methanol (HPLC grade) were obtained from Rathburn (Walkerburn, Scotland). Trifluoroacetic acid (TFA) and dimethyl sulfoxide (DMSO) were from Fisher (Loughborough, UK). Milli-Q system (Millipore, Watford, UK) was used for water purification. XTT sodium salt, menadione sodium bisulfite (2-methyl-1,4-naphthoquinone sodium bisulfite), ciprofloxacin and clarithromycin were from Sigma (Poole, UK). Nutrient broth and agar, Mueller-Hinton agar and phosphate buffered saline (PBS) were from Oxoid (Fisher, Loughborough, UK).

### 3.2. Bacterial Strains

Preliminary screening and XTT bioassays were carried using *E. coli* (NCIMB 8797) and *S. aureus* (NCIMB 6571) obtained from the National Collection of Industrial, Marine and Food Bacteria (NCIMB; Aberdeen, UK).

### 3.3. Plant Material and Extraction Procedures

**Figure 6:**
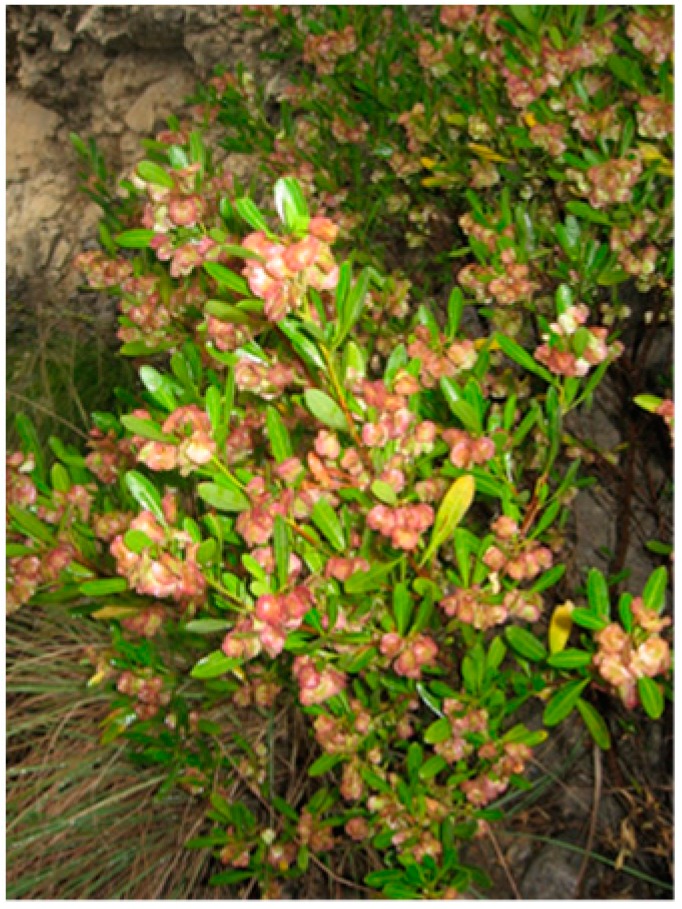
*Dodonaea viscosa* (L.) Jaeq., Florida Hopbush, Hopbush, Varnish plant, Ghwaraskay (*Pushto*), Vilayeti-mehndi (*Hindi*; *Urdu*).

*Dodonaea viscosa* (L.) Jaeq. belongs to the Sapindaceae family. The aerial parts of the plant (Figure 6) were collected from Kohat, Khyber Pakhtoonkhwa (formerly North West Frontier Province; (NWFP)) (Latitude 33°19′59′′ and Longitude 71°10′0′′) Pakistan, in September 2007. A specimen was matched for confirmation of identity with the reference voucher number 592, preserved in the Herbarium of Pakistan (Quaid-i-Azam University, Islamabad, Pakistan). Shade-dried aerial parts (leaves and stems weighing 9 kg) of *D. viscosa* were ground to a fine powder and extracted with ethanol (80% *v*/*v*). The pulverized material was filtered using Whatman No. 1 (460 × 570 mm; approximately 11 µm pore size) in a percolator. Temperature during processing was maintained below 30 °C. Three portions of the percolate were collected at intervals of 96 h finally resulting in 11 L of extract. The ethanol was evaporated using a rotary evaporator (Buchi, Flawil, Switzerland) to give 2.5 L of crude extract. Crude aqueous fraction was sequentially partitioned with *n*-hexane (3 × 1 L), dichloromethane (3 × 1 L), ethyl acetate (3 × 1 L) and *n*-butanol (3 × 1 L) and combined extractions were dried by rotary evaporation [20].

### 3.4. Chemical Characterization

#### 3.4.1. Preparative HPLC

Preparative HPLC was performed using a Biotage Parallex Flex (Biotage, Cardiff, UK) where Flex V3 software was used for instrument control and data acquisition. Separations were performed on a Hyperprep HS C18 column (10 mm I.D. (internal diameter) × 150 mm long; 8 µm particle size; Thermo scientific, Cheshire, UK) with a pre column (KR 100-13 C18-10 CP; Hichrom Ltd., Berkshire, UK). Mobile phase included Milli-Q water (A), and methanol (B). The crude samples were separated using a gradient increasing from 10% to 100% B over 50 min at a flow rate of 10 mL/min. The concentration of crude fractions was 0.2 g/mL, and each injection volume was 0.25 mL. Eluent was monitored at 220 and 254 nm and fractionation was volume-based, collecting 9 mL fractions into deep well microtitre plates (24 × 10 mL; Whatman, Kent, UK). The butanol extract of *D. viscosa* had three fractions (18, 19 and 20) with moderate antibacterial activity (50%–75% reduction in bacterial growth), while six fractions from the hexane extract inhibited only *S. aureus* (>75% reduction in bacterial activity) (Figure 2 and Table 2). Using the data available on the percentage of methanol eluting the bioactive fractions, shorter (30 min) focused gradients were selected for further purification of all the primary bioactive fractions, as summarized for each fraction in Table 4.

**Table 4:** Bioactive fractions from extracts of *D. viscosa* separated by preparative HPLC.

Sample—Fraction (mg Dry Weight)	Percentage Methanol	Focused Gradient
*D. viscosa*—B18 ^a^ (1.0)	42	25%–55% methanol
*D. viscosa*—B19 (2.2)	43	
*D. viscosa*—B20 (5.9)	45	
*D. viscosa*—H42 ^b^ (1.9)	83	80%–100%
*D. viscosa*—H44 (2.9)	86	
*D. viscosa*—H47 (1.6)	91	
*D. viscosa*—H50 (1.1)	97	
*D. viscosa*—H51 (1.0)	98	
*D. viscosa*—H52 (1.6)	100	80%–100% and hold for 10 min

^a^ Fractions from crude butanol extracts (B); ^b^ Fractions from crude hexane (H) extract.

##### Focused Separation of Bioactive Fractions

Fractions from the generic separations that exhibited antibacterial activity were dried, weighed and re-dissolved in 0.5 mL methanol. These were further purified using polarity focused gradients based on the proportion of methanol in which they were eluted during the generic gradient (Table 2). Column and solvents were as described in Section 3.4.1 with an injection volume of 0.25 mL. Fraction collection was based on volume (9 mL fraction).

A total of nine bioactive fractions from *D. viscosa* were further purified using focused gradients over 30 min. Each separation resulted in 35 fractions which were screened by the rapid XTT assay (Figure 2). The five primary fractions from the hexane extract from *D. viscosa* resulted in 22 fractions (Table 5) which inhibited *S. aureus*, out of 18 as active as to the positive control clarithromycin, indicating the potential of this plant as a source of new drugs or drug scaffolds. Slight or no activity was observed in *E. coli* (Figure 2).

**Table 5:** Summary of bioactive sub-fractions from focused preparative HPLC runs of fractions from *D. viscosa* hexane fraction.

Sample—Primary Fraction	Sub-Fraction
*D. viscosa*—H42	12, 14, 21–24, 29, 30
*D. viscosa*—H44	15, 17
*D. viscosa*—H50	25–27
*D. viscosa*—H51	27–30
*D. viscosa*—H52	21, 23, 24, 37, 38

#### 3.4.2. Analytical HPLC

The HPLC system consisted of a Waters Alliance 2695 with a 2996 photodiode array detector (PDA) and a ZQ 2000 mass spectrometer (Elstree, Hertfordshire, UK). A Sunfire C18 column (Length 150 mm × 2.1 mm I.D., particle size 5 µm) which was maintained at 40 °C was used for separation. Mobile phase was Milli-Q water (A) and acetonitrile (B) both containing 0.05% TFA. A gradient increasing from 5% to 100% B over 30 min at a flow rate of 0.3 mL/min was used for separation of samples (10 µL). Eluent was monitored from 210 to 400 nm with a resolution 1.2 nm and by positive ion electrospray (ESI+) in series, scanning from *m*/*z* 100 to 1600 with a scan time of 2 s and inter-scan delay of 0.1 s. Ion source parameters: Sprayer voltage, 3.07 KV; cone voltage, 40 V; desolvation temperature, 300 °C; source temperature, 100 °C. Instrument control, data acquisition and processing were done using MassLynx v4.1 (Waters, Elstree, Hertfordshire, UK). The active component in the *n*-hexane extract of *D. viscosa* was found in H42 (Figure 1) and sub-fraction 12 (Figure 2), where the relative purity (PDA—210 to 400 nm) achieved was >90% (Figure 3).

#### 3.4.3. Physical and Spectral Data for Compound **1**

6β-hydroxy-15,16-epoxy-5β, 8β, 9β, 10α-cleroda-3, 13(16), 14-trien-18-oic acid:

Crystalline solid (20 mg): UV (λ_max,_ MeOH) = 217.7 nm. HR-EIMS *m*/*z* = 332.1879 (calcd. for C_20_H_28_O_4_, 332.2013). The ^1^H-NMR (400 MHz) and ^13^C-NMR (100 MHz) were recorded in MeOD-*d*_4_ on a BRUKER Ultrashield 400 (Bruker UK Limited, Coventry, UK) (Table 2).

### 3.5. Antibacterial Activity

#### 3.5.1. Preliminary Antibacterial Screening

Crude extracts (16.7 mg/mL) were re-suspended in methanol and sterilized using Millex, 33 mm filter (0.22 µm pore size; Millipore, Watford, UK). Aliquots (6 µL) were applied to sterile disks (6 mm diameter punched from Whatman No. 1 filters). Negative control disks had same volume of methanol on each disk. Ciprofloxacin (10 µg/disk) and clarithromycin (15 µg/disk) served as the positive controls for *E. coli* NCIMB 8797 and *S. aureus* NCIMB 6571, respectively. The bacteria were kept on nutrient agar and fresh standardized inocula, turbidity equivalent to 0.5 McFarland (≈1.5 × 10^8^ CFU/mL) [21], were used for each experiment. Disks containing the crude extracts, negative control disks (methanol) and positive controls were aseptically placed on the seeded plates. Prior to overnight incubation at 37 °C, seeded plates with disks were kept in inverted position in refrigerator for 2 h to allow adequate diffusion of extracts. All the tests were performed in triplicate.

#### 3.5.2. XTT Bioassay

XTT solution (1 mg/mL) in PBS was prepared and sterilized using Millex, 33 mm filter (0.22 µm pore size; Millipore, Watford, UK) and stored at −80 °C in cryotubes (Cole-Parmer, London, UK) until use. Menadione (173 μg/mL) in acetone was prepared fresh before to every assay. XTT aliquots were thawed at room temperature and, then, menadione was added to XTT (1:12). From each fraction collected during prep-HPLC, an aliquot (300 µL) was placed in glass vials (2 mL; Alltech, Carnforth, UK) and dried overnight in a laminar flow cabinet and re-suspended in 10% *v*/*v* DMSO/PBS (400 µL) [22]. Samples (100 µL) were transferred into the wells of 96-well flat bottom microtiter plates (Sterilin, Aberbargoed, UK). *E. coli* NCIMB 8797 and *S. aureus* NCIMB 6571 inocula were prepared as mentioned earlier. Inoculum (100 µL) of each test bacteria was added to each of the test well, into negative control (DMSO/PBS) and positive control (ciprofloxacin for *E. coli*, and clarithromycin for *S. aureus*) wells. XTT/menadione mixture (18 µL) was added to each test well and plates were gently shaken. Absorbance at 490 nm was recorded using a BioTek microplate reader (Fisher, Loughborough, UK) at the start of the test and after overnight incubation at 37 °C. All tests were performed in duplicate. Percent increase in absorbance (X) was calculated using formula 1, where A_tN_ and A_t0_ represented mean absorbance values after 24 h incubation and at the start of test, respectively. Similarly, percentage increase in negative control (N) was also calculated using Equation (1). Percent reduction (R) in cell metabolism was calculated using Equation (2), where X_N_ and T_N_ represented percent increase in absorbance of test material and negative control, respectively, at time (N) which was standardized at 24 h.


X_tN_ = A_tN_ − A_t0_/A_t0_ × 100 (1)

R = 100 − ((X_N_/T_N_) × 100) (2)

Fractions were considered to have an antibacterial effect when they gave a mean (*n* = 2) reduction in the XTT colorimetric bioassay of ≥50% (threshold level for antibacterial activity), and <50% value was termed to have slight or no antibacterial activity [13,23].

##### Determination of Minimum Inhibitory Concentration (MIC) of Purified Compound

MIC is defined as the lowest antimicrobial concentration that completely inhibits visible bacterial growth, as detected visually or via an automated or semiautomated method. One hundred µL of purified compound solution (256 µg/mL) in DMSO/PBS was transferred aseptically to the microtiter plates and, sequentially, half diluted in sterile solution of DMSO/PBS such that final dilution of 2 µg/mL was achieved. Then 100 µL of standardized inoculum (≈1.5 × 10^8^ CFU/mL) was added to each of the test wells and into negative control (DMSO/PBS) and positive control (ciprofloxacin for *E. coli* and clarithromycin for *S. aureus*) containing wells. Then, to each test well, 18 µL of XTT/menadione reagent were added and plates were gently shaken. Absorbance at 490 nm was recorded using microplate reader at the start of the test and, then, after 24 h of incubation at 37 °C. The tests were run in duplicate. The wells having no visible bacterial growth, detected visually and having no change in absorbance before and after 24 h of application of test defined the MIC of the compound.

#### 3.5.3. Determination of Minimum Bactericidal Concentration (MBC) of Purified Compound

The MBC was defined as the concentration of the antimicrobial agent that results in the 99.9% reduction in CFU/mL, compared with the organism concentration in the original inocula. The plates used for the evaluation of MIC were utilized such that, after 24 h incubation, the wells that were visibly clear and showed no metabolic activity of bacteria, ascertained from the absorbance and % reduction in bacterial population readings (evaluated using formulas given in Section 3.5.2.), were selected and 50 µL aliquots from each well were spread separately on the entire surfaces of sterile nutrient agar plates with the help of sterile glass spreader, and incubated in inverted position overnight at 37 °C. The tests were run in duplicate. The aliquots of wells that gave no growth defined the MBC of the compound.

## 4. Conclusions

The combination of automated preparative HPLC with simple generic gradients followed by focused gradients combined with the XTT bioassay led to the identification of sub-fractions with slight antibacterial activity. This approach resulted in isolation of a clerodane type diterpenoid, 6β-hydroxy-15,16-epoxy-5β, 8β, 9β, 10α-celoda-3, 13(16), 14-trien-18-oic acid from *D. viscosa*, with MIC of 64 and 128 µg/mL, and MBC of 64 and 256 µg/mL against *S. aureus* and *E. coli*, respectively. This supports the use of *D. viscosa* as antibacterial remedy in traditional systems of healing. Further evaluations of antibacterial spectrum are required. Although only one compound was isolated during this study, there were clearly several bioactive compounds which require further investigation. Moreover, the approach applied in identification and standardization of active metabolite can be capitalized in similar research.

## Supplementary Materials

Supplementary materials can be found at http://www.mdpi.com/1422-0067/16/09/20290/s1.
