# Supplementary material for: Rapid Bioassay-Guided Isolation of Antibacterial Clerodane Type Diterpenoid from Dodonaea viscosa (L.) Jaeq
Source: Int J Mol Sci. 2015 Aug 27;16(9):20290–307. doi: 10.3390/ijms160920290 (PMC4613204; doi:10.3390/ijms160920290)
Supplement: Supplementary file 1 [file ijms-16-20290-s001.pdf]

Supplementary Information

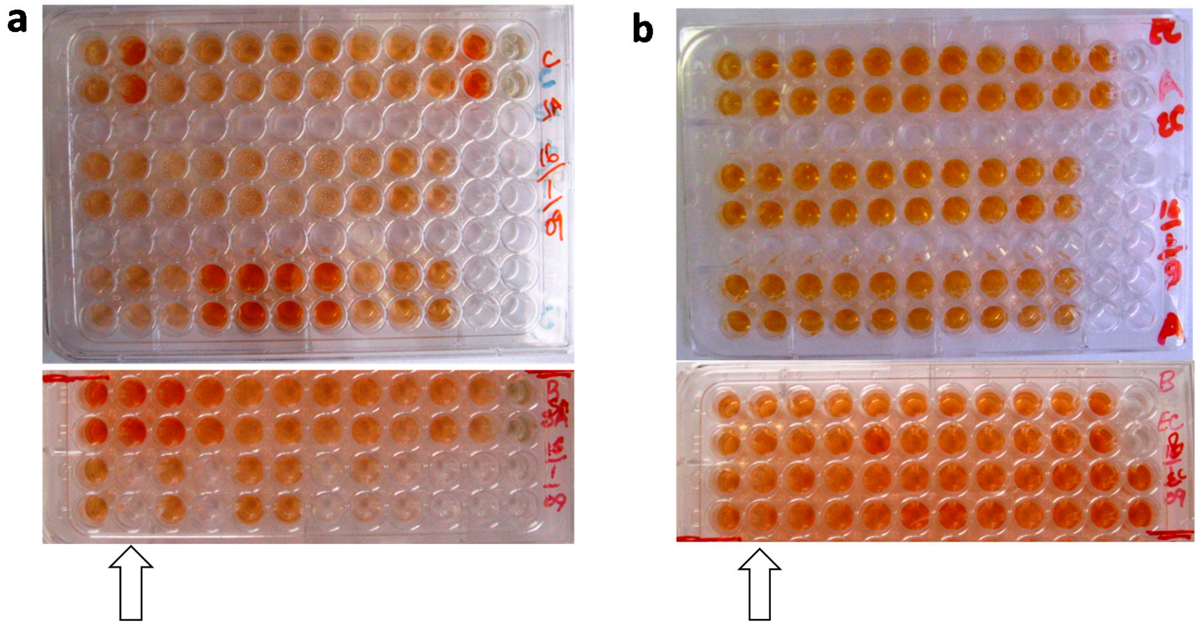

Figure S1. XTT bioassays of fractions obtained after preparative separation of *n*-hexane extract of *D. viscosa* using generic gradient and volume-based fractionation against *S. aureus* (NCIMB 6571) (**a**) and *E. coli* (NCIMB 8797) (**b**). Fraction 42 (arrow) contained high purity, bioactive compound.

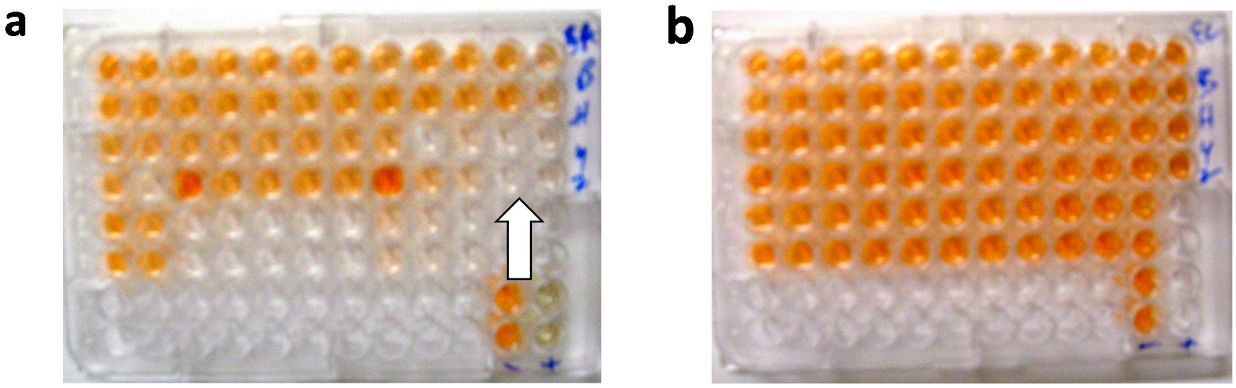

Figure S2. XTT bioassays of sub-fractions from bioactive fraction 42 from *n*-hexane extract of *D. viscosa* using polarity focused gradient against *S. aureus* (NCIMB 6571) (**a**) and *E. coli* (NCIMB 8797) (**b**). Sub-fraction 12 (arrow) contained high purity, bioactive compound.
